# Development and Assessment of a Geographic Knowledge-Based Model for Mapping Suitable Areas for Rift Valley Fever Transmission in Eastern Africa

**DOI:** 10.1371/journal.pntd.0004999

**Published:** 2016-09-15

**Authors:** Annelise Tran, Carlène Trevennec, Julius Lutwama, Joseph Sserugga, Marie Gély, Claudia Pittiglio, Julio Pinto, Véronique Chevalier

**Affiliations:** 1 CIRAD, UPR AGIRs, Ste-Clotilde, Reunion Island; 2 CIRAD, UMR TETIS, Ste-Clotilde, Reunion Island; 3 Food and Agriculture Organization of the United Nations, Rome, Italy; 4 Uganda Virus Research Institute, Entebbe, Uganda; 5 Uganda Ministry of Agriculture, Animal Industry and Fisheries, Entebbe, Uganda; 6 CIRAD, UPR AGIRs, Montpellier, France; University of Queensland, AUSTRALIA

## Abstract

Rift Valley fever (RVF), a mosquito-borne disease affecting ruminants and humans, is one of the most important viral zoonoses in Africa. The objective of the present study was to develop a geographic knowledge-based method to map the areas suitable for RVF amplification and RVF spread in four East African countries, namely, Kenya, Tanzania, Uganda and Ethiopia, and to assess the predictive accuracy of the model using livestock outbreak data from Kenya and Tanzania. Risk factors and their relative importance regarding RVF amplification and spread were identified from a literature review. A numerical weight was calculated for each risk factor using an analytical hierarchy process. The corresponding geographic data were collected, standardized and combined based on a weighted linear combination to produce maps of the suitability for RVF transmission. The accuracy of the resulting maps was assessed using RVF outbreak locations in livestock reported in Kenya and Tanzania between 1998 and 2012 and the ROC curve analysis. Our results confirmed the capacity of the geographic information system-based multi-criteria evaluation method to synthesize available scientific knowledge and to accurately map (AUC = 0.786; 95% CI [0.730–0.842]) the spatial heterogeneity of RVF suitability in East Africa. This approach provides users with a straightforward and easy update of the maps according to data availability or the further development of scientific knowledge.

## Author Summary

Rift Valley fever (RVF) is a zoonotic disease affecting ruminants and humans. It occurs mostly in Africa, causing human deaths and important economic losses in the livestock sector. The RVF virus (RVFV) is transmitted from ruminant to ruminant by mosquitoes. Different climatic, environmental, and socio-economic factors may impact the transmission of the virus. Our work uses all current available knowledge on the epidemiology of the disease and geographic data to map areas suitable for RVFV. The study area includes four East African countries: Kenya, Tanzania, Uganda, three countries which have been historically affected by RVF, and Ethiopia, where the disease has never been reported but which shares borders with infected countries. The resulting maps are compared with the locations of outbreaks reported in livestock. Our results demonstrate the capacity of the spatial multi-criteria evaluation method to map with accuracy the areas suitable for RVF occurrence. Thus, the method we developed provides users with risk maps that could be used for early warning detection and implementation of control measures.

## Introduction

Caused by a Phlebovirus (Bunyaviridae) that affects both humans and livestock, Rift Valley fever (RVF) is considered to be one of the most important viral zoonoses in Africa. The RVF virus (RVFV) is transmitted from ruminant to ruminant by mosquitoes [1]. Although never demonstrated, there is field, serological and virological evidence of transmission without any use of vectors [2], suggesting an alternative transmission of the RVFV between ruminants through direct contact. Humans become infected mainly through direct contact with ruminant viremic fluids, such as blood or abortion products, but also through mosquito bites.

Although in the majority of human cases RVF infection is asymptomatic or causes mild illness, severe forms are characterized by retinitis, encephalitis or hemorrhagic fever. In ruminants, RVF infection causes abortion storms in groups or flocks of pregnant females and acute deaths in newborns [3]. Both health and economic impacts can be greatly reduced when control measures, such as vaccination, insecticide spraying and dissemination of information, are quickly implemented. The delay between case detection and control measure implementation depends on, among other factors, the efficiency of surveillance networks; therefore, an accurate definition of at-risk areas needs to be monitored along with other factors.

RVF virus circulation has been reported in several eco-climatic areas: arid in western Africa and the Arabic Peninsula [4, 5]; sub-humid in East Africa [6, 7]; wet forests in central Africa [8]; dam and irrigated agricultural land under hot climatic conditions in Egypt, Mauritania and Sudan [9–11]; and humid highlands in Madagascar [2, 12].

Depending on the areas of concern, different risk factors have been identified, either for transmission, spread or human and/or livestock occurrence. Potential mosquito vectors of the RVFV belong to the genera *Aedes*, *Anopheles*, *Culex*, *Eretmapodites* and *Mansonia*. The majority of the factors driving mosquito vector presence and abundance, thus driving the risk of RVF transmission, are related to climate, water and landscape. The *Aedes* genus is mostly associated with temporary water bodies such as flooded area, temporary pond, puddles, and rice fields. *Culex* and *Anopheles* mosquito breeding areas are diverse and could be temporary (rice fields, swamps) or permanent (lakes, ponds) bodies of water. Stagnant and permanent water bodies are the habitats of *Eretmapodites* and *Mansonia*, respectively [13].

In fact, the presence of temporary water bodies and floodplains are outbreak risk factors for RVF in semi-arid areas in eastern Africa, the Arabian Peninsula and western Africa [4]. In eastern and southern Africa, the risk of RVFV infection has been shown to vary as a function of rainfall, temperature, and a remotely sensed vegetation index (NDVI: normalized difference vegetation index) [14, 15]. Artificial water bodies, such as dams and irrigated rice fields, are also known to be associated with the high abundance of RVFV vectors in western Africa [4]. In addition to eco-climatic factors, cattle density has been identified as a risk factor for transmission of the RVFV [6, 16]. Habitat, gender, profession, and contact with ruminants and ruminant products have also been identified as risk factors of RVF occurrence in humans [17].

The Horn of Africa has been historically affected by RVF [18]. However, the occurrence of RVF has never been reported in Ethiopia, which shares borders with infected countries, namely, Kenya [6], northern Somalia [19], and Sudan [20]. In Uganda, although no outbreak in humans or animals were reported until 2016, a recent serological survey revealed that RVFV was endemic in goats in four districts [21].

In Kenya and Tanzania, where RVF is endemic, historical knowledge indicates that ‘dambos’ are areas at risk of RVF [22]. Moreover, recent eco-epidemiological studies identified the main environmental risk factors for RVF, which has allowed for health targeted surveillance by health authorities [6, 14, 23]. However, the application of these models to regions outside of the study area of interest may lead to incorrect inferences. Moreover, information related to the suitability of both Ethiopia and Uganda ecosystems for the transmission of the RVFV are scarce. Given this lack of information, pragmatic approaches must be developed to provide risk maps that could be used for early warning detection and implementation of control measures.

Spatial multi-criteria evaluation (MCE) is a rapid and pragmatic knowledge-based method adapted for mapping disease suitability in the absence of large epidemiological datasets. Defined as ‘a process that transforms and combines geographical data and value judgments (expert and bibliographic knowledge, including uncertainties, subjective and qualitative information) to obtain appropriate and useful information for decision making’ [24], this method has been used to map suitable areas for RVF transmission in Africa, on a continental scale [25] and in Senegal [26], and in the European countries of Spain [27] and Italy [28]. However, the predicted maps could not be validated for European countries which are disease-free regions, while in Senegal the validation could only be performed in a qualitative way [26]. Moreover, in these studies, only the ‘amplification step’, defined as the local transmission of the RVFV to its hosts by mosquito vectors, was considered and did not account for the possible transportation of the virus from a primary outbreak to secondary foci in a ‘spread step’. This process may involve different risk factors than those of the amplification step, such as animal trade [12, 29].

The goal of the present study was to adapt a geographic knowledge-based method [25] to identify suitable areas for RVF amplification and spread in four Eastern African countries, namely, Kenya, Tanzania, Uganda and Ethiopia, and to assess the predictive accuracy of the model using livestock outbreak data from Kenya and Tanzania.

## Materials and Methods

### Epidemiological definitions

Under suitable conditions and after the introduction or low-level transmission of a given pathogen, the pathogen can be locally transmitted to a ‘primary’ host through direct or vectorial transmission and then transferred from the primary infectious host to several secondary hosts; this is the “amplification” process [30]. Therefore, ‘spread’ is defined as the transportation of the pathogen from the primary outbreak to secondary foci. In this study, ‘suitability’ is defined as the ability of a habitat to support either the amplification or the spread of RVF. Amplification is a necessary condition for primary RVF occurrence. Both amplification and spread are needed for secondary outbreaks.

### Identification of risk factors from bibliographic review

Following the spatial multi-criteria evaluation (MCE) methodology that has been detailed elsewhere [25, 31], we first identified the amplification and spread risk factors of RVF through a literature review. PubMed and ISI Web of Knowledge were searched for articles published from 1980 to 2014 using the search terms ‘‘rift valley fever” AND (separately) ‘‘model” or ‘‘spatial”, or “risk factors” or “analysis” using the ‘‘all fields” option to allow for the retrieval of articles in which the search terms appeared in the titles, abstracts, or keywords. Inclusion criteria were reviews and/or articles using expert knowledge, and/or statistical and mathematical modelling approaches to model RVF risk to ruminants or humans. A total of 62 references were thus included.

In Table 1, we listed the factors associated with the risks of amplification and spread of RVF according to the published literature review. Only risk factors that could be mapped were selected for the risk mapping process. The following risk factors were thus included:

**Table 1:** Risk factors associated with the amplification and spread of Rift Valley fever in livestock populations as identified by the published literature review.

	Risk factor^a^ *[proxy*^b^*]*	Reference
**Individual level**	Species	[4, 40–43]
	Age	[12, 16, 29, 44, 45, 46]
**Production systems**	Sheep, goat and cattle densities* [Water bodies as zones of increased contact among animals]*	[16, 32]
**Markets and trade**	Proximity to animal markets*	[12, 29]
	Ruminant trade [roads, railways]*	[19, 32–34, 47, 48]
	Traditional and commercial practices	[12, 29]
	Festival periods	[32, 49, 50]
**Wildlife**	Presence of wild ruminants [proximity to conservation areas]*	[35–39]
**Vector populations and landscape variables**	*Aedes*, *Culex*, *Mansonia* and *Anopheles* genera [Elevation* and landform*, land cover*, soil type*]	[6, 7, 12, 16, 46, 51–73]
**Climate**	Season	[14, 22, 32, 71, 74, 75, 76]
**Surveillance**	Quarantine, surveillance system efficiency/sensitivity (protective factors)	[14, 50, 74, 77]

^a^ Risk factors included in the present study (= risk factors for which spatial data or proxies were available) are indicated by an asterisk

^b^ If the data corresponding to an identified risk factor was not available, a proxy (= variable assumed to resemble the risk factor) was used

#### Sheep, goat and cattle densities

Assuming the existence of direct transmission, an increase in domestic ruminant density is expected to increase the number of potentially infectious contacts that a susceptible individual experiences over a given time; therefore, there is a greater risk of amplification. Because infectious ruminants may travel after being sold, ruminant density was also considered as a factor of spread [16, 32].

#### Markets and trade

The role of ruminant trade in RVFV dissemination was previously documented [19, 29, 32–34]; increased densities of markets, roads and railways are expected to be associated with increased ruminant trade and thus related to a high risk of RVF spread.

#### Wildlife

A detailed literature review of the role of a wild mammal reservoir in the epidemiologic cycle of RVFV concluded that buffaloes and other wild ruminants could contribute to the amplification and spread of the disease in southern and eastern Africa [35–39]. The proximity of wildlife national parks and the existence of domestic and wild ruminants with overlapping home ranges could thus be associated with increased risk of RVF amplification and spread.

#### Vector populations and landscape variables

RVF occurrence is expected to be strongly associated with the distribution and the abundance of the RVFV vector populations [14, 22]. Environmental variables (land cover, elevation, landform, soil type) have been shown to be associated with increased incidence of RVF in humans in Kenya and were hypothesized to provide optimal vector habitat at each life stage, thus reflecting suitable conditions for the densities of RVFV vectors [6]. Moreover, due to the limited active flying capacities of mosquito vectors, a shorter distance from water bodies is expected to be associated with an increase in the risk of amplification (vector-borne transmission is associated with permanent and temporal water bodies providing breeding sites for the RVFV vectors, such as ‘dambos’ in Kenya [22]) and spread (increased contact among animals that share rivers, lakes or ponds as sources of drinking water).

### Generation of standardized geographical layers for each risk factor

A search was conducted to obtain digital geographical data for each identified risk factor (Table 1). The different sources of the data that were used and their main characteristics are provided in S1 File and S1 Table. The data were imported into a geographic information system (GIS) and processed to produce standardized spatial risk factor layers, namely a mosquito index (reflecting the suitability for RVF mosquito vectors), sheep density, goat density, cattle density, proximity to markets, road density, railways density, proximity to water bodies, proximity to wildlife national parks (software: ESRI ArcMap and ArcMap Spatial Analyst Extension, Redlands, CA, USA). At the end of the process, each image layer was raster-based, with pixel dimensions of 300 m x 300 m. The scale of all spatial risk factor layers was continuous, ranging from 0 (completely unsuitable) to 1 (completely suitable). The different sources of the data used and the calculation method of the standardized geographical layers are provided in S1 File. The resulting maps for the risk factor layers are presented in S1 Fig.

### Generation of weights for each risk factor

We assumed that the weight of each risk factor in the amplification and spread processes were not equivalent. For example, small ruminants are known to be more susceptible than cattle for the transmission of the RVFV [42] and, therefore, more prone to play a more important role during the amplification phase than the latter. Markets are aggregation points where ruminant herds meet and have potential contact with each other before returning back to their living area; markets are, therefore, more important for spread than roads that may be used by herders for travelling. Based on the literature review and our own expertise, we ranked RVF risk factors for virus amplification and spread according to their putative relative importance in both processes [78]. Factors were compared two at a time: 1) We first specified whether risk factor A was more or less important than risk factor B; and 2) We specified the degree of importance of factor A regarding factor B on a nine-point scale using the Saaty scale (factor A can be extremely more important, very strongly more important, strongly more important, moderately more important, equally important, moderately less important, strongly less important, very strongly less important or extremely less important than factor B), resulting in a pair-wise comparison matrix. A numerical weight was then derived for each risk factor from the pair-wise comparison matrix [79]. We calculated pair-wise comparison matrices separately for amplification and spread, considering the vector distribution being much more important in the amplification process than in the virus spread phase.

### Combination of the risk factors and creation of suitability maps

Then, three different maps were generated, considering two distinct groups of risk factors and their associated weights for the amplification and spread steps.

Assuming that vector presence is a necessary condition for RVF amplification, the suitability of areas for RVF amplification was calculated for each raster cell as the product of the mosquito index map (computed as described in S1 File) and a weighted linear combination (WLC) of each of the standardized geographical risk factor layers associated with RVF amplification using its corresponding weight. Regarding the spread process, the raster maps for RVF risk factors associated with RVF spread were combined by computing a WLC with their corresponding weights.

The suitability maps for amplification and spread were then combined to create two different suitability maps for RVF occurrence. First, the values of suitability indices for amplification and spread were recoded in three classes (low/medium/high suitability) using a quantile discretization. These two recoded maps were then merged into a primary synthetic RVF suitability map with nine classes corresponding to all possible combination of amplification suitability (low/medium/high) and spread suitability (low/medium/high). Second, the areas with the highest risk (suitability estimates greater than 0.1, i.e., in the 90^th^ percentile) of RVF amplification were selected. The Euclidean distance between these areas was calculated and transformed into a ‘proximity to RVF amplification areas’ index, which assumed a sigmoid-shaped decreasing relationship between 0 and 100 km and negligible risk thereafter. Finally, suitability estimates for RVF occurrence, combining the spread and amplification processes, were computed as the product of the suitability estimates for RVF spread and the proximity to RVF amplification areas index, resulting in a second synthetic RVF suitability map expressed as a continuous suitability index.

### Sensitivity and uncertainty analyses

A sensitivity analysis (SA) was conducted to assess the sensitivity of the method to the expert choices. To determine the effect of a change in the weights applied to each risk factor, a range of weight values to explore was defined by adding and subtracting 25% from the original weights. Ten weight values within this range were tested (+/-5%, +/- 10%, +/-15%, +/-20% and +/-25%). Each of the newly calculated weights was incorporated into the modelling process, while other factor weights were proportionally decreased or increased such that the sum of the weights equaled 1. For each combination of weights obtained, maps of suitability indices for RVF amplification, spread and occurrence were calculated, and a total of 169 suitability maps for RVF occurrence were generated. Based on these different realizations, the contribution of the different risk factor weights to model output variance was calculated for each country (see S2 File for details).

Finally, an uncertainty surface was produced. It represented the standard deviation of the different suitability maps resulting from the change in weights [80].

### Validation

**Fig 1:**
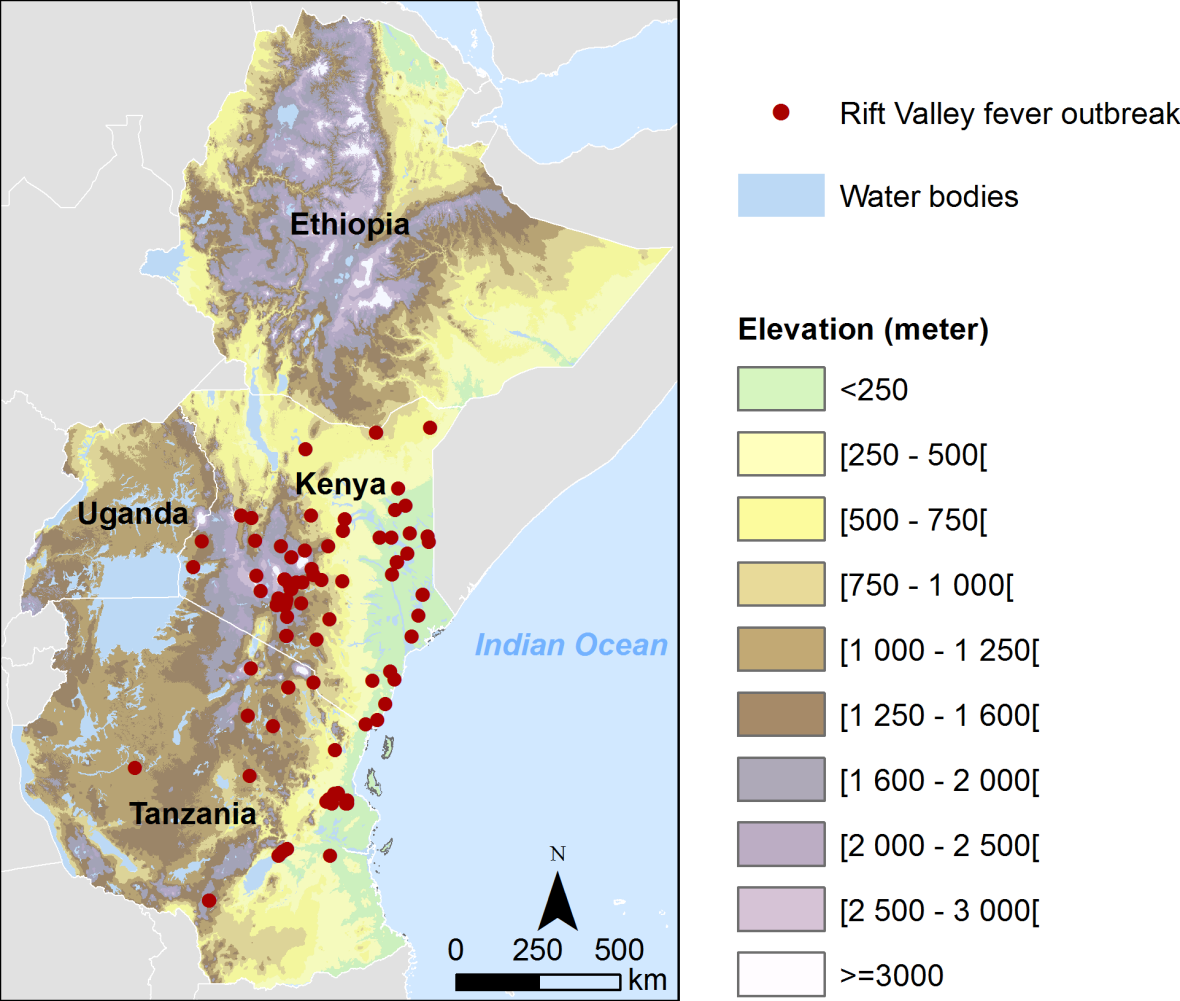
Location of Rift Valley fever outbreaks in livestock reported in Kenya and Tanzania between 1998 and 2012.

RVF outbreaks in livestock reported in Kenya and Tanzania between 1998 and 2012 were collected to assess the consistency of RVF suitability map (Source: FAO EMPRES-i database: http://empres-i.fao.org). A total of 145 outbreaks were located using geographic coordinates (Fig 1).

Then, 150 locations of disease ‘pseudoabsence’ data were randomly generated in these two countries, under the condition of being 25 km from another ‘pseudoabsence’ or outbreak location. The value of the quantitative suitability estimates for RVF occurrence was extracted for each ‘presence’ or ‘pseudoabsence’ location and the AUC (area under curve) of the ROC curve [81], and the sensitivity and specificity were calculated to evaluate the quality of the suitability map.

## Results

### Weights of RVFV amplification and spread risk factors

The resulting weights of risk factors for RVFV amplification and spread are presented in Table 2 (see S1 and S2 Tables for the details of the pair-wise comparison matrices).

**Table 2:** Risk factor weights calculated using the analytical hierarchy process, regarding RVFV amplification and spread processes.

Risk factor	Weight RVF amplification	Weight RVF spread
**Mosquito index**		0.025
**Sheep density**	0.288	0.204
**Goat density**	0.288	0.204
**Cattle density**	0.203	0.144
**Proximity to markets**	0.080	0.175
**Density of roads**	0.042	0.062
**Proximity to water bodies**	0.034	0.062
**Density of railways**	0.034	0.062
**Proximity to wildlife national parks**	0.031	0.062

Regarding amplification, we assumed that the mosquito index was the most important factor, and a necessary condition for RVFV amplification. Then, small ruminant densities were identified as important factors, followed by (in descending order) cattle density, proximity to markets that are aggregation points for animals, proximity to roads, water bodies and railways, and proximity to wildlife parks.

Regarding spread, we considered that viremic hosts were the most important means of virus dissemination and that markets were, again, an aggregation point for people and their herds. The proximity to roads and water bodies were also important factors because they allow for trade movements. Finally, proximity to wildlife national parks and the mosquito index were factors of low influence in the spread process.

### Suitability maps for RVF amplification, spread and occurrence

**Fig 2:**
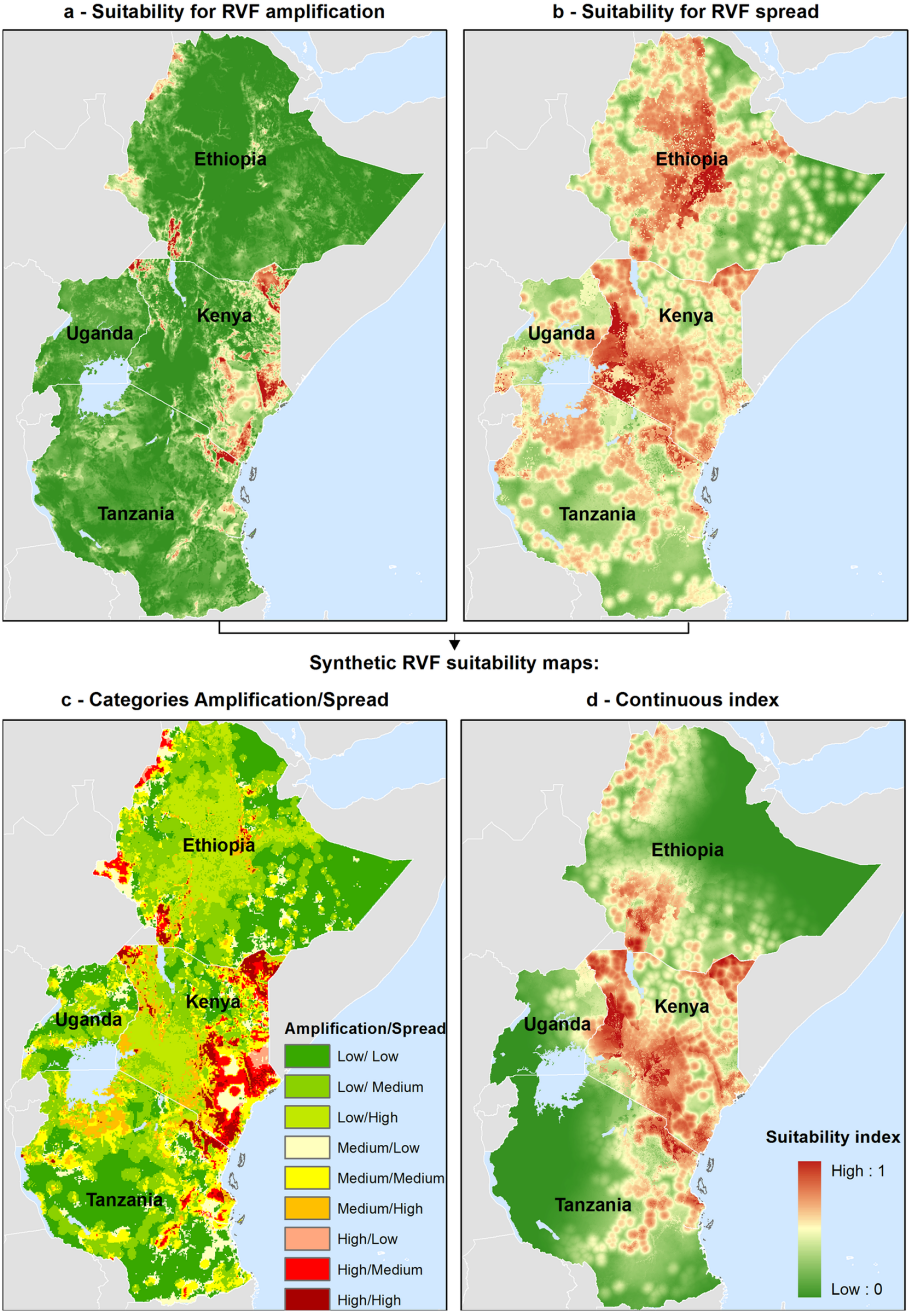
Suitability maps for RVF amplification (a) and spread (b) are combined to produce a synthetic suitability map for RVF occurrence in livestock with nine classes corresponding to all possible combinations of amplification suitability (low/medium/high) and spread suitability (low/medium/high) (c) and a second synthetic RVF suitability map expressed as a continuous suitability index (d).

Fig 2 presents the different maps produced from the MCE process: a map of suitability for RVF virus amplification (Fig 2a), a map of suitability for RVF spread (Fig 2b) and a final map of suitability for RVF occurrence in domestic ruminants (Fig 2c) (maps of all standardized risk factors are provided in S1 Fig).

According to the results of the MCE, areas suitable for RVF amplification were located in the low elevation areas of Kenya (the eastern coast, the northeastern portion that borders Ethiopia and Somaliland, and, to a lesser extent, the northwest region bordering Uganda), Tanzania (northeastern portion) and Ethiopia (in the Northwest and Southwest). Uganda presented a very low suitability for RVF amplification in domestic ruminants.

In addition, the suitability map for RVF spread in domestic ruminants (Fig 2b) showed a different pattern, identifying the highlands of Ethiopia, Kenya, and Uganda as areas favorable to RVF spread. In Tanzania, areas suitable for RVF spread were located in the northern part of the country.

The combination of the ‘amplification’ and ‘spread’ maps resulted in two final synthetic maps of the areas suitable for RVF occurrence in domestic ruminants that were complementary (Fig 2c and 2d). According to the map that highlights the different categories of amplification/spread combinations (Fig 2c), the majority of eastern Kenya was identified as highly suitable for RVF occurrence, with a medium-to-high suitability for RVF amplification combined with medium-to-high suitability for RVF spread. This pattern was also observed in northeastern Tanzania and southwestern Ethiopia. Areas with medium suitability for both RVF amplification and spread, such as northwestern Tanzania, or areas with low amplification suitability but high spread suitability, such as western Kenya, the majority of Uganda and the Ethiopian highlands, were identified as suitable for RVF occurrence. This first map also highlighted areas with low suitability for RVF occurrence (low suitability for amplification and spread), such as eastern Ethiopia and central Tanzania. Taking into account the proximity of the areas with the highest suitability for RVF amplification, the second synthetic RVF suitability map (Fig 2d) highlighted the different patterns of the areas suitable for RVF occurrence: the majority of Kenya was identified as suitable; however, in the three other countries, the areas suitable for RVF occurrence were smaller than those shown in Fig 2c.

### Sensitivity and uncertainty analysis

The uncertainty of the surface-based model produced by the data for the four countries showed that the predictions of the location of suitable areas for RVF occurrence in livestock were robust, meaning that they remained stable when varying the risk factor weights in the ‘amplification’ and ‘spread’ steps. Indeed, the maximum standard deviation (STD) of the suitability maps for RVF occurrence was less than 0.1. The results highlighted a spatial heterogeneity in uncertainty, with higher uncertainty in the western parts of Ethiopia and Kenya (S2 Fig).

**Fig 3:**
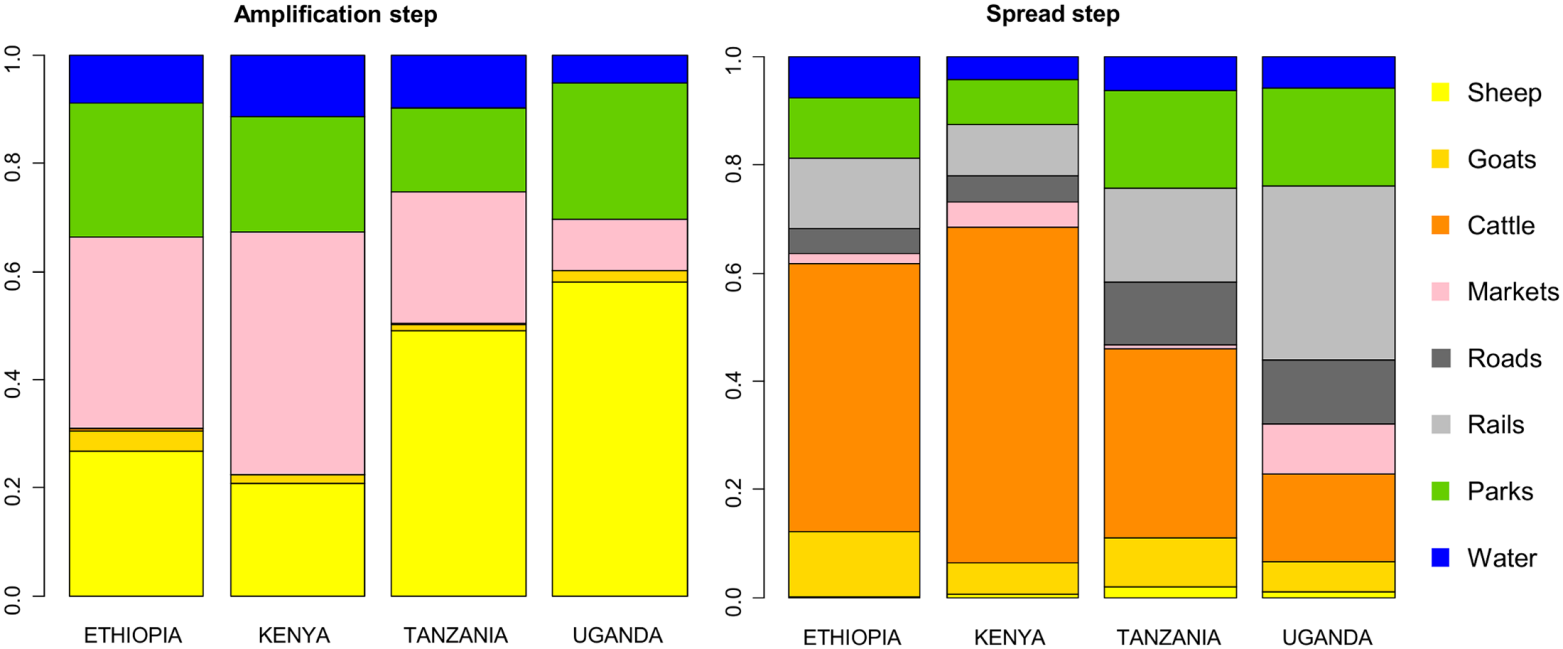
Contribution of the different risk factor weights to model output variance.

The sensitivity analysis showed that the variation in the suitability index was explained by four factors for the amplification step and by seven factors for spread (Fig 3). The most sensitive parameters for the amplification step were the sheep density and the proximity to markets, wildlife national parks, and water bodies. Regarding the spread step, the most sensitive parameters were the cattle and goat densities, the density of roads and railways, and the proximity to national parks and water bodies. The importance of these sensitive parameters varied among the four countries, particularly the importance of the livestock densities (cattle, sheep and goats) (Fig 3).

### Map validation

**Fig 4:**
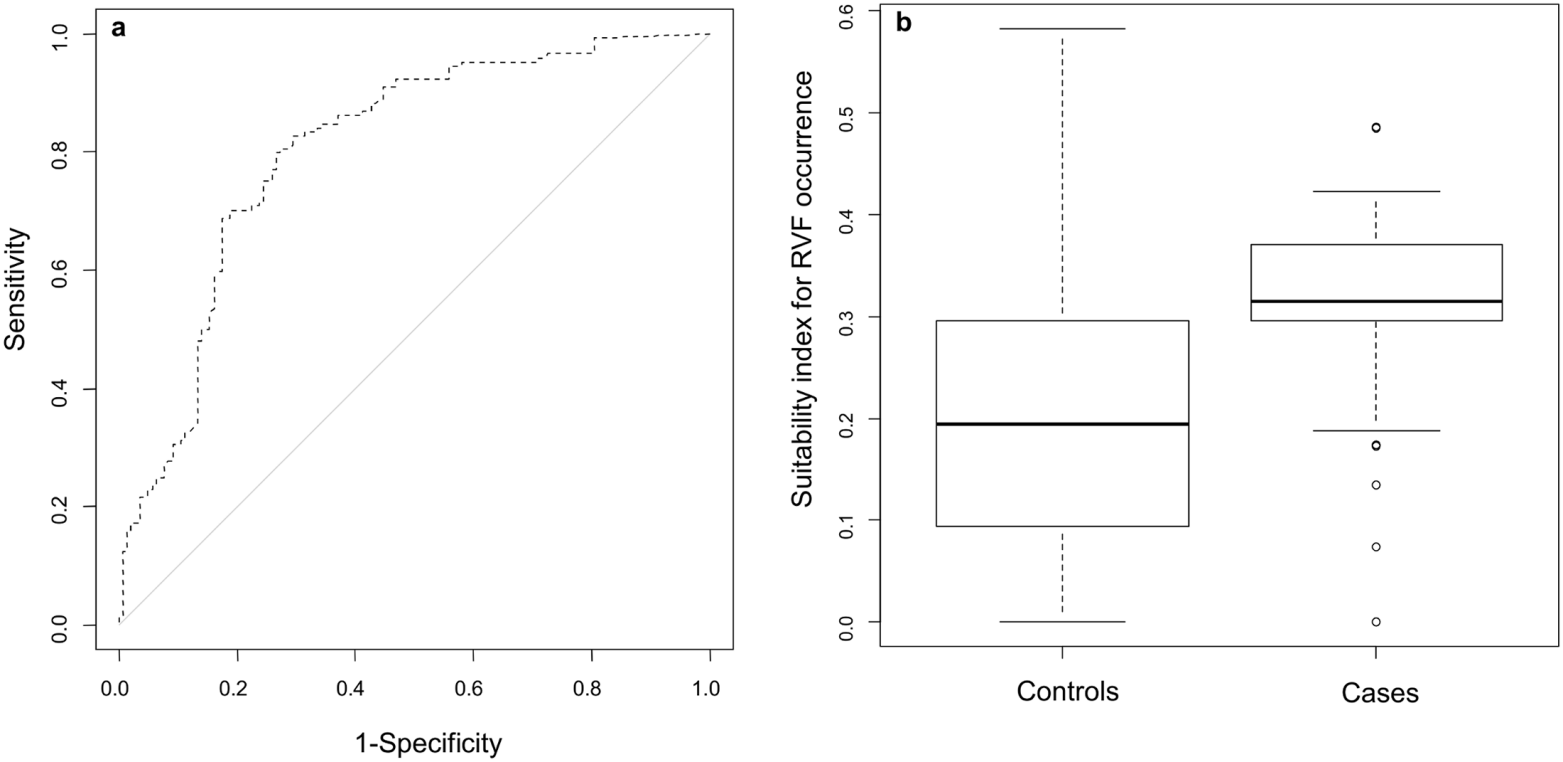
Assessment of the suitability index for RVF occurrence in livestock in Kenya and Tanzania. a) ROC curve. b) Box-plot showing RVF occurrence suitability index values for cases (RVF outbreak locations) and controls (random ‘pseudoabsence’ locations). Box-plots show median values (solid horizontal line), 50^th^ percentile values (box-plot outline), 90^th^ percentile values (whiskers), and outlier values (open circles).

**Fig 5:**
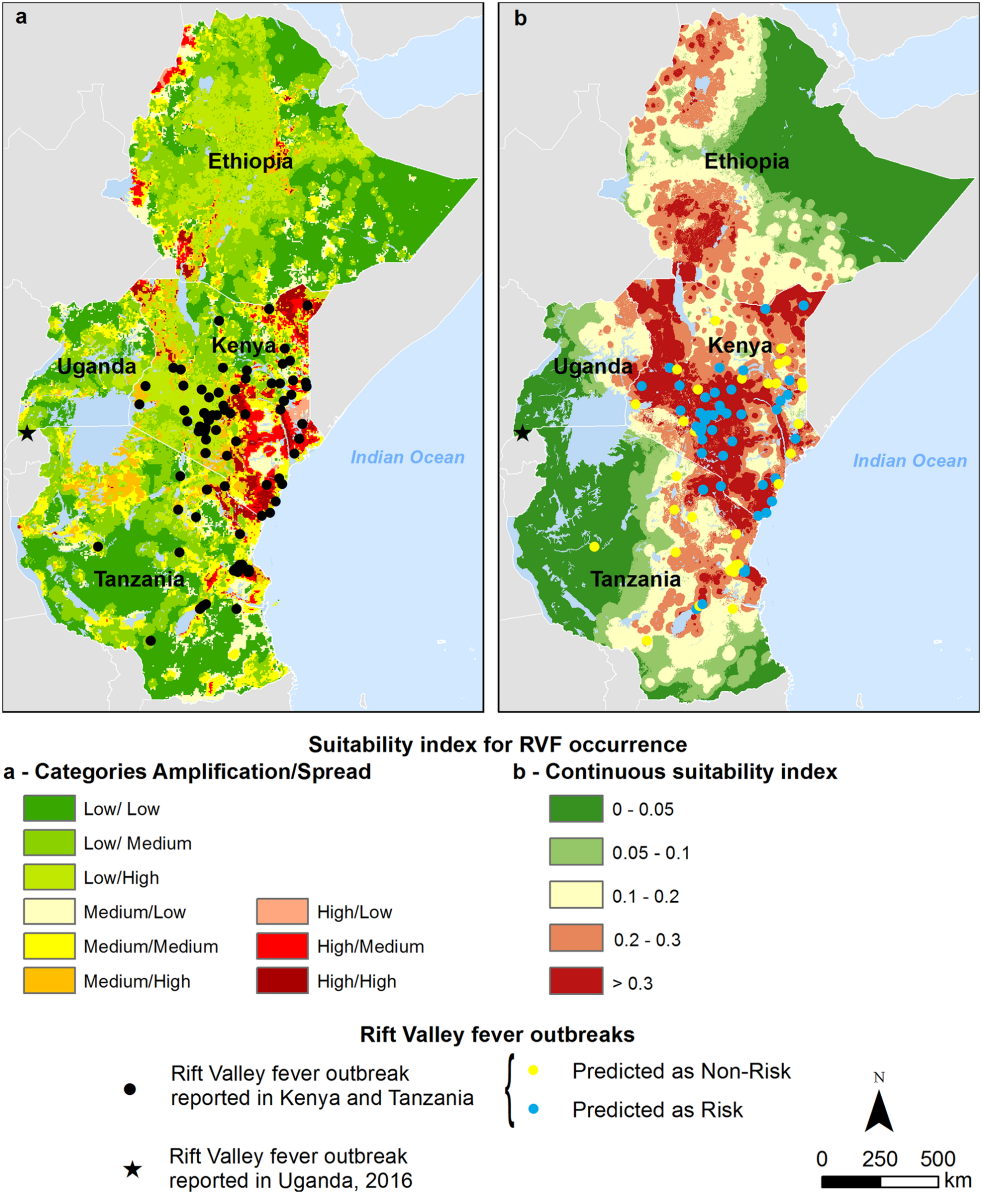
Suitability maps for RVF occurrence and the locations of RVF outbreaks in livestock: a) Combination of amplification and spread suitability categories; (b) continuous suitability index. Blue dots indicate the outbreak locations that were identified in the RVF at-risk areas. Yellow dots indicate outbreak locations not considered to be at-risk.

The ROC AUC associated with the suitability map for RVF occurrence in Kenya and Tanzania showed a good fitting (AUC = 0.786; 95% CI [0.730–0.842]) (Fig 4a), demonstrating the capacity of the model to distinguish ‘presence’ from ‘absence’ locations with good predictive accuracy (Fig 4b). With a cut-off point of 0.3 maximizing both sensitivity and specificity, the sensitivity was 0.74, and the specificity was 0.75. A total of 74% (107 out of 145) of the RVF outbreak locations were mapped in at-risk areas, which were defined as the areas with a suitability index for RVF occurrence greater than 0.3, the cut-off point value maximizing sensitivity and specificity (Fig 5b).

## Discussion

Many regions from Kenya and Tanzania were previously and heavily affected by RVF outbreaks [6, 82]. However, some areas may be at-risk without having experienced outbreaks in past years. The identification of these areas is essential for implementing risk-based surveillance and reducing the impact of RVF human and animal outbreaks in the coming years. Until 2016, Uganda and Ethiopia remained free from outbreaks, but their geographical locations as well as the livestock exchanges they have with their neighbors make these two countries highly vulnerable to the disease.

In this context, the implementation of the GIS-based MCE method for RVF risk mapping appeared to be a very efficient method to map suitability areas for the amplification and spread of the virus based on freely available geographic data. To our knowledge, this is the first study aiming to produce regional suitability maps for RVF using MCE methodology combined with outbreak dataset validation.

Validation of the suitability map using disease presence and background data randomly generated produced good results according to the ROC AUC method (AUC = 0.786). However, the use of randomly generated ‘pseudoabsence locations’ may be controversial; indeed, the absence of reported outbreaks is not an evidence of absence of pathogen transmission. The results of regional serological surveys may give a more precise evaluation of the RVF suitability map.

Nevertheless, 74% of the reported RVF outbreaks in livestock were located in areas with the highest predicted suitability for RVF occurrence (Fig 5b). Interestingly our MCE-based model performed better than other predictive models based purely on climatic anomalies and previously validated with human outbreaks [14]. These models, which showed the highest accuracy in the Eastern African region, included 65% of the human case locations in predicted at-risk areas. Two human cases of RVF have been reported in early March 2016 in the Kabale District, southwestern Uganda [83]. The outbreak occurred in an area that was identified by our model as poorly suitable for RVF amplification but highly suitable for RVF spread (Fig 5a). This result is highly consistent with the socio-economic and ecological environment of Kabale district. Indeed, Kabale is an important commercial center with six animal markets, a situation associated with a higher risk for RVF spread according to our assumptions. Being outside of the ‘potential epizootic area mask’ [14], this area is not predicted by the climate-based model [83]. Despite the strong 2015–2016 El Niño phenomenon and the associated abnormal rainy season in East Africa, no substantial climatic anomalies were observed in the Kabale area during the 2016 epidemics. Differently from the southeastern and central districts in Uganda and neighboring countries, such as Kenya and Tanzania, both cumulative precipitation as well as NDVI values were lower than or equal to average in Kabale area during the period September 2015 to February 2016, except for short periods in October and December 2015. We therefore hypothesize a little role of vector-borne transmission in the Kabale outbreak.

Thus, our results highlighted the importance of taking into account livestock data and the factor of animal trade in addition to environmental factors to develop predictive maps of RVF occurrence. Moreover, these maps increase the confidence level for the approach applied to RVF free-areas [27, 28].

Indeed, the MCE approach we applied to four countries of eastern Africa was very similar to previous modelling studies that used the same approach in different geographic contexts [25–28]. All of these studies considered two main categories of risk factors: on the one hand, those related to domestic ruminant densities, and on the other hand, those related to vector presence (*i*.*e*., vector distributions or proxies of vector distributions, such as temperature, elevation, rainfall, and proximity to aquatic areas). One of the distinctive features of our study was the ability to distinguish RVF amplification and spread steps in the modelling process, thus considering risk factors related to animal trade and movements (markets, roads and railways). Moreover, the hypothesized role of wildlife reservoirs in the amplification and spread of RVF was considered.

Identifying areas of low amplification with high spread suitability and vice versa (Fig 5a) was expected because these two epidemiological phenomena imply different mechanisms: vector and host densities favor local amplification, whereas animal movements favor the long-range spread of the disease. From a control perspective, surveillance strategies should be adapted; active surveillance in sentinel herds would be relevant in amplification areas that act as virus sources for areas that are not at risk of amplification, and analysis of the trade network and the existing links between amplification areas and other regions could be used as an early warning tool to protect spread areas from viremic ruminant introduction in case of primary foci.

However, the limits of our method must be noted. First, in the absence of homogeneous information on RVFV vector abundance and distribution in our study area, we used environmental variables to map a vector index reflecting the suitability of locations for the presence of RVF vectors. These variables were identified through a study performed in Kenya; this study may not be perfectly relevant for the three other countries because this vector suitability map needs to be validated by landscape-targeted mosquito trappings in each country. Among mosquito species vectors recorded in the countries of concern, several were demonstrated to be competent in the lab [42]. However, even if competency measures were to provide elements to infer the potential role of a given mosquito species in RVF outbreaks, these measures are not sufficient to definitely incriminate the species. Indeed, mosquito abundance and foraging behavior are major elements that also shape the epidemiology of arboviruses. Better knowledge of these entomological characteristics should be considered to improve the vector index map.

Second, the spatial scale chosen for mapping the suitable areas for disease transmission has a great impact on the produced maps due to the spatial resolution of the data used to calculate the risk factors and the choice of the risk factors included to map suitability areas for pathogen transmission. Moreover, the weight attributed to each risk factor may differ between regional and national scales. In this study, we provided regional maps of suitability for RVF; however, maps produced on a national scale with higher spatial resolution, derived from risk factors and weights discussed with experts of each country (with particular attention paid to the most sensitive weights identified by the SA), would be more accurate and useful for surveillance and control purposes. Moreover, the threshold used to define the areas highly suitable for virus amplification (90^th^ percentile threshold considering the whole study area) may introduce a bias in suitability predictions. This threshold value should be adapted for each country to provide better predictions at the national level.

Lastly, limitations of the produced map are related to the availability of data used as risk factors and their quality. For instance, due to a lack of geographic data on the locations of veterinary services and surveillance networks within each country, this information was not taken into account in our model, although both are key factors for the control of animal diseases—the spread intensity and magnitude from primary foci depending on the detection delay and, thus, on the efficiencies of veterinary and surveillance services. Moreover, the immunological status of ruminant populations induced by previous virus circulation episodes or vaccination campaigns were not taken into account although they are important factors, as demonstrated in South Africa in 2010 [84]. Additional information on how landscape features and socio-economic factors impact domestic and wild ruminant movements may also be important to refine the cost distance calculations of markets, water and wildlife park proximity indices.

In this study, we focused on mapping the areas suitable for RVF amplification and spread; thus, we focused on the spatial dimension of RVF risk. Although transmitted by mosquitoes and probably by direct contact, RVF is a seasonal disease, occurring during or at the end of the rainy season when mosquito abundance is at its highest. Future work should take into account this temporality to provide seasonal suitability maps for RVF transmission in livestock. Coupling climate-based models [14] with the RVF suitability map, which includes livestock and commercial variables, would allow for the development of seasonal suitability maps for RVF transmission in livestock. However, it must be stressed that this requires a good understanding of the drivers of RVF emergence. Indeed, with the exception of Kenya, where a strong association was demonstrated between heavy rainfall events and outbreak occurrence [22], rainfall may not be the only key factor for RVF emergence. Host density, associated with suitable climatic conditions and the introduction of the virus by ruminant trade, probably led to the 2000 outbreak in Yemen [32]. In Madagascar in 2008, no abnormal rainfall was noticed before the outbreak [85]. In Senegal in 2003, an intense transmission was described without any abnormal rainfall [44]. Soti *et al*. (2012) observed that in this region, the rainfall pattern rather than rainfall abundance could be responsible for triggering outbreaks [86]. Therefore, the seasonality of outbreaks should be incorporated in models with caution, depending on the area considered.

## Conclusion

The present study confirmed the capacity of GIS-based MCE method to synthesize available scientific knowledge and map with accuracy the spatial heterogeneity of RVF suitability in four countries of East Africa. Moreover, such an approach enables users a straightforward and easy updating of the maps according to data availability or scientific knowledge development to include more precise geographic data or additional risk factors and to modify the weights of each factor.

## Supporting Information

S1 Fig Standardized risk factors for RVF amplification and spread in Ethiopia, Kenya, Uganda and Tanzania. Values of each risk factor range from zero (low risk: black areas) to one (high risk: white areas).
(TIF)

S2 Fig Uncertainty map (standard deviation of RVF occurrence suitability maps) for Ethiopia, Kenya, Uganda, and Tanzania. (TIF)

S1 Table Details of the Geographic Information Systems manipulations required to convert the collected data into risk factor layers in raster format. (PDF)

S2 Table Scaling functions applied to Rift Valley fever risk factor layers. (PDF)

S3 Table Correlation matrix for risk factors associated with Rift Valley fever amplification and spread. (PDF)

S4 Table Pair-wise comparison matrices of the analytical hierarchy process (AHP) for risk factors associated with Rift Valley fever amplification. (PDF)

S5 Table Pair-wise comparison matrix of the analytical hierarchy process (AHP) for risk factors associated with Rift Valley fever spread. (PDF)

S1 File Spatial risk factors for Rift Valley fever: Sources of the data used, and calculation of the corresponding standardized geographical layers. (PDF)

S2 File Sensitivity analysis. (PDF)
