# Supplementary figures and images for: Development and Assessment of a Geographic Knowledge-Based Model for Mapping Suitable Areas for Rift Valley Fever Transmission in Eastern Africa
Source: PLoS Negl Trop Dis. 2016 Sep 15;10(9):e0004999. doi: 10.1371/journal.pntd.0004999 (PMC5025187; doi:10.1371/journal.pntd.0004999)

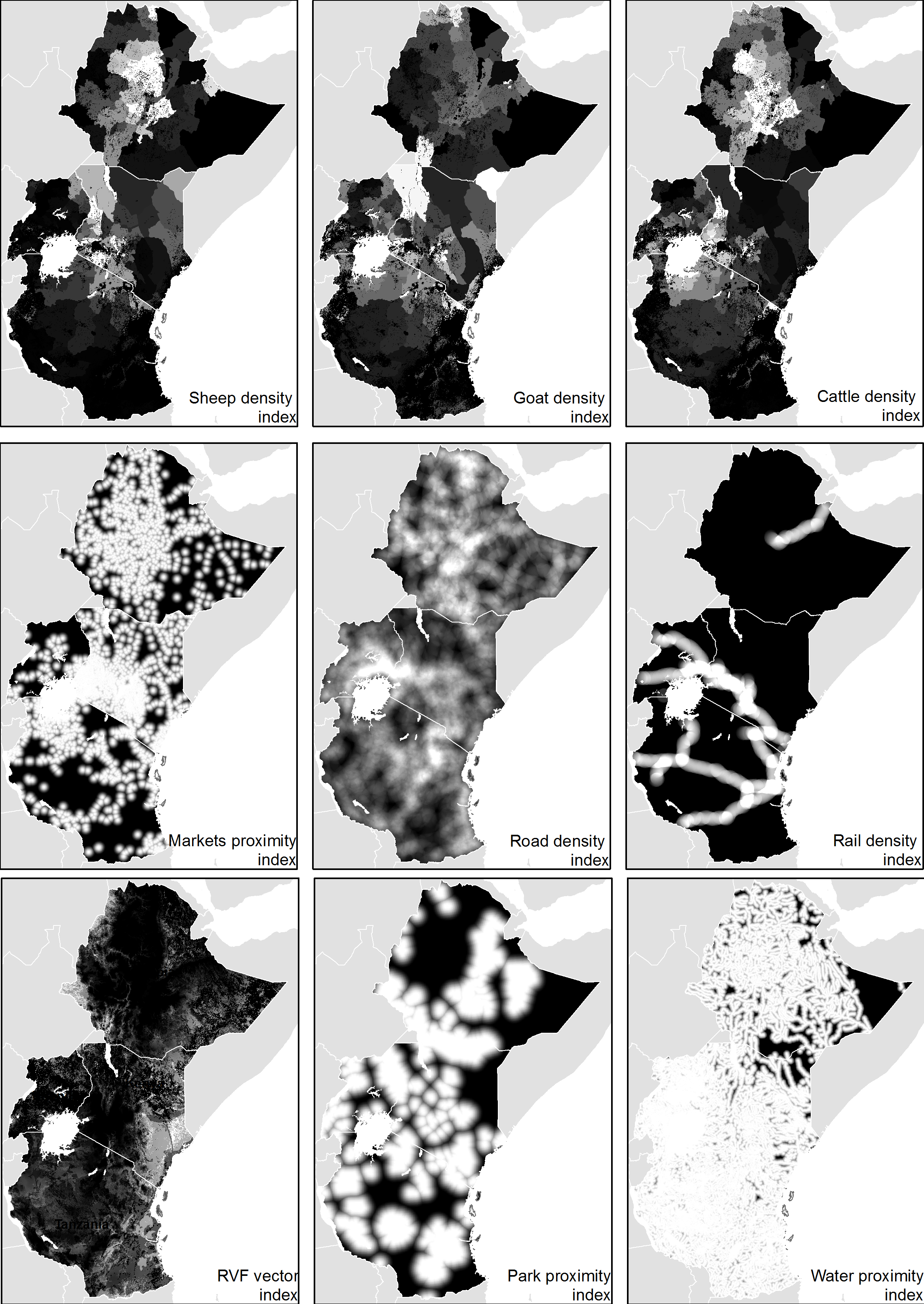

Supplement: S1 Fig — Values of each risk factor range from zero (low risk: black areas) to one (high risk: white areas). (TIF) [file pntd.0004999.s001.tif]

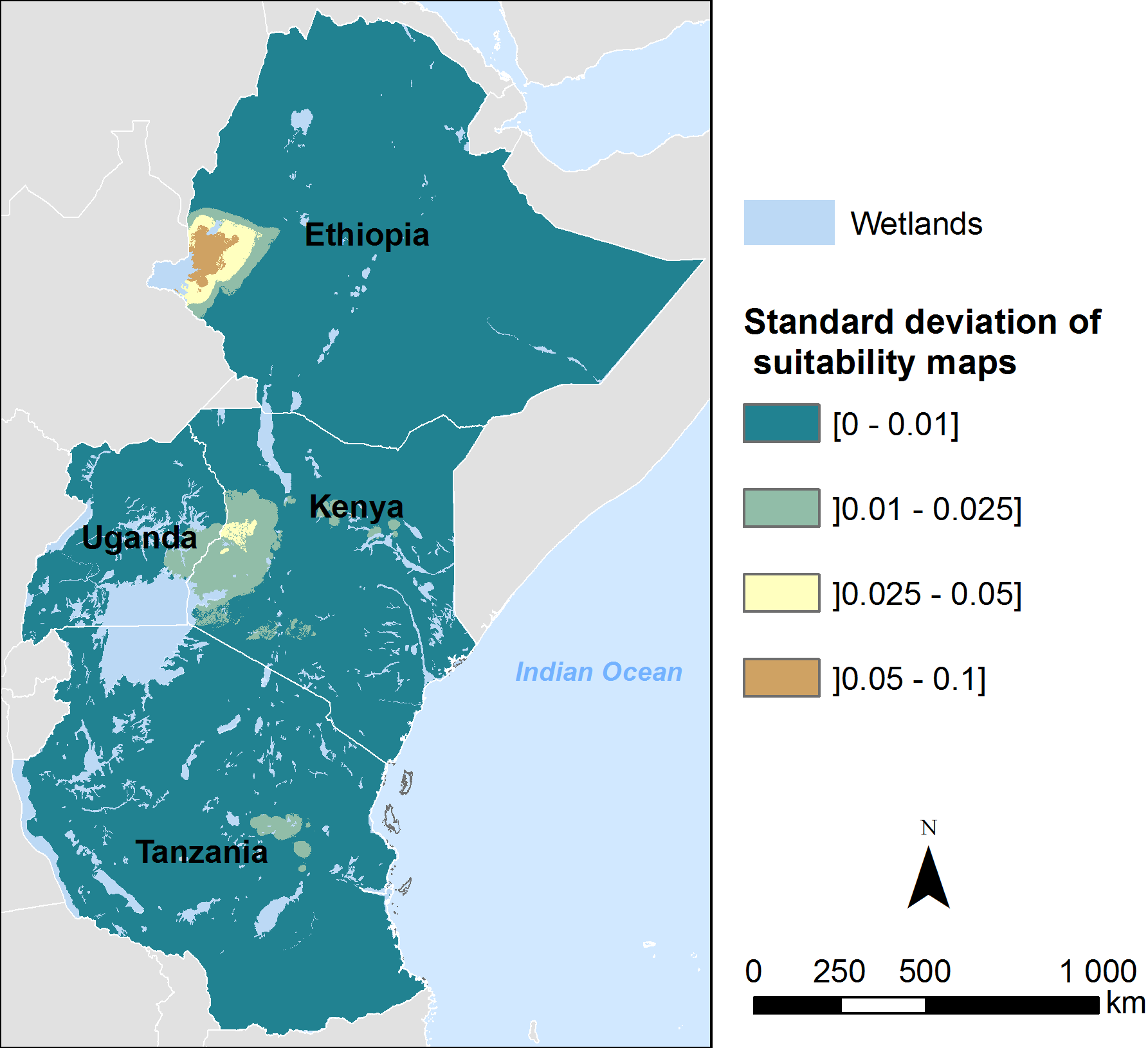

Supplement: S2 Fig — (TIF) [file pntd.0004999.s002.tif]
