# Supplementary material for: Development and Assessment of a Geographic Knowledge-Based Model for Mapping Suitable Areas for Rift Valley Fever Transmission in Eastern Africa
Source: PLoS Negl Trop Dis. 2016 Sep 15;10(9):e0004999. doi: 10.1371/journal.pntd.0004999 (PMC5025187; doi:10.1371/journal.pntd.0004999)
Supplement: S1 Table — (PDF) [file pntd.0004999.s003.pdf]

S1 Table: Details of the Geographic Information Systems manipulations required to convert the collected data into risk factor layers in raster format

**Collected data associated to RVF suitability**	**GIS manipulation**	**Risk factor raster**	
Districts (polygons)<br>Table with number of sheep per district	Join geographic layer and table.<br>Calculate animal densities (nb animal/km²).	Sheep density	
Districts (polygons)<br>Table with number of goats per district		Goat density	
Districts (polygons)<br>Table with number of cattle per district		Cattle density	
Small ruminants' markets of Uganda, Ethiopia, and Kenya (points)	Calculate and map distance (km) to markets	Distance to markets	
Population map<br>(resolution 0.000833333° ~ 900 m at the latitude of the study area)	Calculate and map distance (km) to areas with population densities > 1000 inhab./km², with elevation map as cost map		
Roads (polylines)	Calculate and map density of roads per 100 square kilometres	Density of roads	
Railways (polylines)	Calculate and map density of railways per 100 square kilometres	Density of railways	
Wildlife national parks (polygons)	Calculate and map distance (km) to: Conservation Area, Controlled Hunting Area, Game Controlled Area, Game Reserve, Game sanctuary, Hunting reserve, National Park, National Reserve, Nature Reserve, Sanctuary, Wildlife Reserve. Use elevation map as cost map	Distance to wildlife national parks	
Rivers and wetlands (polylines and polygons)	Calculate and map distance (km) to rivers and wetlands, with elevation map as cost map	Distance to rivers and wetlands	
Elevation map<br>(resolution 90 m x 90 m)	Apply a scaling function (negative linear relationship)	Elevation index	Vector index<br>(Eq. 2)
	Calculate slope and extract plain areas	Plain index	
Land cover map<br>(resolution 300 m x 300 m)	Extract bushy areas and reclassify (1: bush; 0: other land cover types)	Bush index	
Global soil map (polygons)	Extract water retaining soils (1: water retaining soils; 0: other soil types)	Soil index	
