# Supplementary material for: Development and Assessment of a Geographic Knowledge-Based Model for Mapping Suitable Areas for Rift Valley Fever Transmission in Eastern Africa
Source: PLoS Negl Trop Dis. 2016 Sep 15;10(9):e0004999. doi: 10.1371/journal.pntd.0004999 (PMC5025187; doi:10.1371/journal.pntd.0004999)
Supplement: S2 Table — (PDF) [file pntd.0004999.s004.pdf]

S2 Table: Scaling functions applied to Rift Valley fever risk factor layers

Risk factor map	Scaling function
Sheep density	Positive linear relationship
Goat density	Positive linear relationship
Cattle density	Positive linear relationship
Proximity to markets	Sigmoidal, monotonically decreasing relationship between 0 and 50 km, with negligible risk after 50 km[1].
Density of roads	Positive linear relationship
Density of railways	Positive linear relationship
Proximity to wildlife national parks	Sigmoidal, monotonically decreasing relationship between 0 and 100 km, with negligible risk after 100 km[1].
Proximity to rivers and wetlands	Sigmoidal, monotonically decreasing relationship between 0 and 50 km, with negligible risk after 50 km[1].
Vector index map	No manipulation required

[1] From expert knowledge on domestic and wild ruminants mobilities
