# Supplementary material for: Development and Assessment of a Geographic Knowledge-Based Model for Mapping Suitable Areas for Rift Valley Fever Transmission in Eastern Africa
Source: PLoS Negl Trop Dis. 2016 Sep 15;10(9):e0004999. doi: 10.1371/journal.pntd.0004999 (PMC5025187; doi:10.1371/journal.pntd.0004999)
Supplement: S3 Table — (PDF) [file pntd.0004999.s005.pdf]

S3 Table. Correlation matrix for risk factors associated with Rift Valley fever amplification and spread.

**Risk factor***	Sheep	Goat	Cattle	Markets	Roads	Rails	Water	Parks	Vector
Sheep	1.00	**0.61**	**0.71**	0.30	0.15	0.06	0.08	0.03	0.18
Goat	0.61	1.00	**0.53**	0.21	0.07	0.02	0.07	0.08	0.02
Cattle	0.71	0.53	1.00	0.28	0.27	0.01	0.13	0.01	0.21
Markets	0.30	0.21	0.28	1.00	0.39	0.19	0.11	0.08	0.13
Roads	0.15	0.07	0.27	0.39	1.00	0.24	0.02	0.08	0.20
Rails	0.06	0.02	0.01	0.19	0.24	1.00	0.15	0.09	0.00
Water	0.08	0.07	0.13	0.11	0.02	0.15	1.00	0.19	0.02
Parks	0.03	0.08	0.01	0.08	0.08	0.09	0.19	1.00	0.07
Vector	0.18	0.02	0.21	0.13	0.20	0.00	0.02	0.07	1.00

* Sheep: sheep density; Goat: goat density; Cattle: cattle density; Markets: proximity to ruminant’s markets; Roads: density of roads; Rivers: proximity to rivers; Railways: density of railways; Parks: proximity to wildlife national parks; Vector: vector index.
