# Supplementary material for: Development and Assessment of a Geographic Knowledge-Based Model for Mapping Suitable Areas for Rift Valley Fever Transmission in Eastern Africa
Source: PLoS Negl Trop Dis. 2016 Sep 15;10(9):e0004999. doi: 10.1371/journal.pntd.0004999 (PMC5025187; doi:10.1371/journal.pntd.0004999)
Supplement: S4 Table — (PDF) [file pntd.0004999.s006.pdf]

S4 Table. Pair-wise comparison matrixes of the Analytical Hierarchy Process (AHP) for risk factors associated with Rift Valley fever amplification.

**Risk factor***	Sheep	Goat	Cattle	Markets	Roads	Rivers	Railways	Parks	**Weight**
Sheep	1	1	3	5	7	7	7	7	**0.288**
Goat		1	3	5	7	7	7	7	**0.288**
Cattle			1	5	7	7	7	7	**0.203**
Markets				1	3	3	3	3	**0.080**
Roads					1	1	1	3	**0.042**
Rivers						1	1	1	**0.034**
Railways							1	1	**0.034**
Parks								1	**0.031**

* Sheep: sheep density; Goat: goat density; Cattle: cattle density; Markets: proximity to ruminant's markets; Roads: density of roads; Rivers: proximity to rivers; Railways: density of railways; Parks: proximity to wildlife national parks. Vector index is not included in the pair-wise matrix as its weight is equal to 1 in Equation 2 (S1 File).
