# Supplementary material for: Development and Assessment of a Geographic Knowledge-Based Model for Mapping Suitable Areas for Rift Valley Fever Transmission in Eastern Africa
Source: PLoS Negl Trop Dis. 2016 Sep 15;10(9):e0004999. doi: 10.1371/journal.pntd.0004999 (PMC5025187; doi:10.1371/journal.pntd.0004999)
Supplement: S5 Table — (PDF) [file pntd.0004999.s007.pdf]

S5 Table. Pair-wise comparison matrix of the Analytical Hierarchy Process (AHP) for risk factors associated with Rift Valley fever spread.

**Risk factor***	Vector	Sheep	Goat	Cattle	Markets	Roads	Rivers	Railways	Parks	**Weight**
Vector	1	1/7	1/7	1/5	1/5	1/3	1/3	1/3	1/3	**0.025**
Sheep		1	1	3	1	3	3	3	3	**0.204**
Goat			1	3	1	3	3	3	3	**0.204**
Cattle				1	1	3	3	3	3	**0.144**
Markets					1	3	3	3	3	**0.175**
Roads						1	1	1	1	**0.062**
Rivers							1	1	1	**0.062**
Railways								1	1	**0.062**
Parks									1	**0.062**

* Vector: vector index; Sheep: sheep density; Goat: goat density; Cattle: cattle density; Markets: proximity to ruminant's markets; Roads: density of roads; Rivers: proximity to rivers; Railways: density of railways; Parks: proximity to wildlife national parks.
