# Supplementary material for: Development and Assessment of a Geographic Knowledge-Based Model for Mapping Suitable Areas for Rift Valley Fever Transmission in Eastern Africa
Source: PLoS Negl Trop Dis. 2016 Sep 15;10(9):e0004999. doi: 10.1371/journal.pntd.0004999 (PMC5025187; doi:10.1371/journal.pntd.0004999)
Supplement: S1 File — (PDF) [file pntd.0004999.s008.pdf]

S1 File. Spatial risk factors for Rift Valley fever: sources of the data used, and calculation of the corresponding standardized geographical layers

Once risk factors have been identified (Table 1), data sources have been identified and the geographic data have been manipulated in order to produce appropriate spatial risk factor layers for inclusion in the model. First, the original data are transformed into raster layers, representing information on risk factors at every point across their extent. All raster layers were resampled to a resolution of 300 m x 300 m, a good compromise between the spatial resolutions of the different datasets and computational limitations due to the size of the study area. Full details on the generation of the risk factor layers in raster format are presented in Table S1. Second, the spatial risk factor rasters have been standardized on a continuous scale, with pixel values ranging from 0 (low suitability) to 1 (high suitability). The fuzzy membership functions used to standardize the spatial risk factor rasters on a 0-1 scale are presented in Table S2. Finally, we assessed the correlation between the different risk factor layers (Table S3). They were not correlated, with the exception of domestic ruminant densities.

Sheep, goat and cattle densities. Livestock densities were obtained from national agriculture statistics at district level [1-4]. Animal densities were standardized assuming a positive linear relationship between animal densities and suitability for RVF amplification and spread. Thus, for each pixel *i* and risk factor *j*, a scaled risk factor value was computed as:

$$x'_{i,j} = \frac{x_{i,j} - \min_j}{\max_j - \min_j} \tag{1}$$

where $x_{i,j}$ is the original value of risk factor *j* at pixel *i*, $x'_{i,j}$ is the scaled value, and min_j and max_j the minimum and maximum values for the risk factor *j*, respectively.

Density of roads and railways. Roads and railways data were obtained from the Digital Chart of the World (DCW). Density values (number of roads / km², number of railways / km²) were computed using Geographic Information System (GIS) tools, and standardized between

0 and 1 assuming a positive linear relationship with the suitability for RVF amplification and spread (Eq. 1).

Proximity to livestock markets, water bodies, and wildlife national parks. The location of small ruminant's markets was provided by national (the National Bureau of Statistics for Uganda) and international organizations (the Food and Agriculture Organization of the United Nations, FAO, for Kenya and Ethiopia). For Tanzania, the human population density (> 1000 inhab./km²) was used as a proxy for the location of livestock markets, after checking of its relevance in the three other countries, using human population estimates from WorldPop project [5]. Water bodies (rivers and wetlands), and wildlife national parks were sourced from public domain databases [6, 7].

An elevation weighted distance to each feature of interest (livestock markets, water bodies, wildlife national parks), was calculated and transformed into a 'proximity to' index assuming a sigmoidal decreasing relationship with the suitability for RVF between 0 and 50 km (as the maximum distance usually travelled by walking herds [8, 9]), and negligible risk thereafter (S2 Table).

Vector suitability index. As a vector-borne disease, RVF occurrence is highly linked to the distribution of the mosquito species acting as RVF vectors. **In Ethiopia**, the presence of the following potential RVF vectors were recorded: *Mansonia uniformis/africana, Culex tritaeniorhynchus, Culex zombaensis, Culex quinquefasciatus, Culex poicilipes, Culex theileri, Culex pipiens, Culex neavei, Aedes mcintoshi, Aedes circumluteolus* (source: Collection d'Arthropodes d'Intérêt Médical, MIVEGEC/IRD, http://www.arim.ird.fr/). **In Kenya**, recent entomological studies showed that the following potential vector mosquito species were present: *Aedes mcintoshi/circumluteolus, Aedes ochraceus, Mansonia uniformis, Culex poicilipes, Culex bitaeniorhynchus, Anopheles squamosus, Mansonia africana, Culex quinquefasciatus, Culex univittatus, Aedes pembaensis, Culex univittatus, and*

Culex Bitaeniorhynchus. According to several authors, different mosquito species may serve as epizootic/ epidemic vectors of RVFV in diverse ecological contexts, creating a complex epidemiologic pattern in East Africa [10, 11]. **In Tanzania**, very few information is available. Among potential RVF vectors, recent study reported the presence of the Culex complex and *Aedes aegypti* (capable of transmitting the virus in laboratory, found naturally infected in Sudan [12, 13]. **In Uganda**, RVFV isolation has been recorded in three species, namely *Aedes tarsalis* (Smithburn 1948), *Mansonia uniformis* (Williams 1960) and *Aedes africanus* (Weinbren & Mason 1957). Three other species may be considered as potential vectors because of their abundance: *Aedes simpsoni*, *Aedes dendrophilus* and *Aedes aegypti*.

In absence of homogeneous information on RVF vector abundance and distribution through the four countries, we used environmental variables to map a vector index with values ranging from 0 to 1, reflecting an index of suitability for the presence of RVF vectors. Environmental variables were chosen based on the results of an eco-epidemiological study on RVF in Kenya [10], identifying elevation, landcover (densely bushy areas), soil type and plain areas, as risk factors for RVF occurrence, all of them associated with vector abundance. The corresponding raster layers were obtained from different public domain sources: the elevation data from the Shuttle Radar Topographic Mission (spatial resolution 90 m x 90 m) [14], land cover map by the Globcover project (spatial resolution 300 m x 300 m) [15], and the soil data from FAO (provided as vector format, 1:5.000.000 scale) [16]. They were then standardized on a continuous scale (0-1), and combined according to Eq. 2 to produce a vector index raster, with values ranging from 0 to 1.

$$vector\ index = elevation\ index * \frac{bush\ index + soil\ index + plain\ index}{3} \qquad (2)$$

References

1. Ethiopian central statistical agency. Agricultural sample survey. Volume II: Report on livestock and livestock characteristics. Addis Ababa: Federal Democratic Republic of Ethiopia; 2013. 194 p.

2. Ministry of Agriculture Kenya - Animal production division. Provincial summaries of livestock population statistics for 2009: Ministry of Agriculture; 2009.

3. Ministry of Agriculture United Republic of Tanzania. Tanzania national sample census of agriculture 2007/2008. Volume III: Livestock sector - national report2012.

4. Uganda Bureau of Statistics. Uganda national household survey. Report on the agricultural module. Kampala: Uganda Bureau of Statistics; 2002.

5. WorldPop. Population estimates 2014. Available from: http://www.worldpop.org.uk.

6. geonetwork F. AFRICOVER - Rivers and wetlands 2014. Available from: www.fao.org/geonetwork/.

7. UNEP-WCMC. The World Database on Protected Areas (WDPA) [On-line] Cambridge, UK: UNEP-WCMC; 2014. Available from: http://www.protectedplanet.net/.

8. Konandreas P, Anderson F. Cattle herd dynamics: an integer and stochastic model for evaluating production alternatives. Addis Ababa, Ethiopia: International Livestock Centre for Africa; 1982. p. 103.

9. Western D. The Environment and Ecology of Pastoralists in Arid Savannas. Developmenr and Change. 1982; 13:183-211.

10. Hightower A, Kinkade C, Nguku PM, Anyangu A, Mutonga D, Omolo J, et al. Relationship of climate, geography, and geology to the incidence of Rift Valley fever in Kenya during the 2006-2007 outbreak. Am J Trop Med Hyg. 2012 Feb; 86(2):373-80.

11. Sang R, Kioko E, Lutomiah J, Warigia M, Ochieng C, O'Guinn M, et al. Rift Valley fever virus epidemic in Kenya, 2006/2007: the entomologic investigations. Am J Trop Med Hyg. 2010 Aug; 83(2 Suppl):28-37.

12. McIntosh BM, Dickinson DB, dos Santos I. Rift Valley fever. 3. Viraemia in cattle and sheep. 4. The susceptibility of mice and hamsters in relation to transmission of virus by mosquitoes. J S Afr Vet Assoc. 1973 Jun; 44(2):167-9.

13. Mweya CN, Kimera SI, Mellau LS, Mboera LE. Inter-epidemic abundance and distribution of potential mosquito vectors for Rift Valley fever virus in Ngorongoro district, Tanzania. Global health action. 2015; 8:25929.

14. SRTM. Shuttle Radar Topographic Mission 90m Digital Elevation Data 2014. Available from: http://srtm.csi.cgiar.org/.

15. ESA. GlobCover Land Cover Map 2009. Available from: http://due.esrin.esa.int/page_globcover.php.

16. geonetwork F. Digital Soil Map of the World 2014. Available from: http://www.fao.org/geonetwork/srv/en/main.home.
