# Supplementary material for: Development and Assessment of a Geographic Knowledge-Based Model for Mapping Suitable Areas for Rift Valley Fever Transmission in Eastern Africa
Source: PLoS Negl Trop Dis. 2016 Sep 15;10(9):e0004999. doi: 10.1371/journal.pntd.0004999 (PMC5025187; doi:10.1371/journal.pntd.0004999)
Supplement: S2 File — (PDF) [file pntd.0004999.s009.pdf]

S2 File. Sensitivity analysis

A sensitivity analysis was conducted to assess the sensitivity of the method to the expert choices. To determine the effect of a change in the weights applied to each risk factor, the range of weight values to explore was defined by adding and subtracting 25% from the original weights. Ten weight values (w_m) within this range were tested (+/-5%, +/- 10%, +/-15%, +/-20% and +/-25%). Each of the newly calculated weight was incorporated into the modelling process, while other factor weights were proportionally decreased or increased such that the sum of the weights equals 1:

$$w_i = (1 - w_m) * \frac{w_{i0}}{1-w_{m0}},\ 1 \leq \mathrm{i} \leq \mathrm{n}, \mathrm{i} \neq \mathrm{m} \qquad (1)$$

where w_{m0} and w_{i0} are the weights in the base model of the main changing risk factor and of the *i*-th risk factor, respectively.

For each combination of weights obtained, a map of suitability index for RVF amplification, spread and occurrence was computed. A total of 169 suitability maps for RVF occurrence were thus generated.

Based on these different realizations, the contributions of the variation weights to the suitability index variability, averaged at country-level were evaluated using a linear regression [1]. For each aggregated output, a linear regression model was fitted with all the principal effects of the risk factors. The contribution of factor *i* to the variation in output was the ratio of the sum of squares related to *i* on the total sum of squares of the model.

References.

1. Saltelli, A., K. Chan, and E.M. Scott, *Sensitivity Analysis*. 2000, New York, NY, USA: John Wiley and Sons.
